# Controlled Phase and Tunable Magnetism in Ordered Iron Oxide Nanotube Arrays Prepared by Atomic Layer Deposition

**DOI:** 10.1038/srep18401

**Published:** 2016-01-27

**Authors:** Yijun Zhang, Ming Liu, Bin Peng, Ziyao Zhou, Xing Chen, Shu-Ming Yang, Zhuang-De Jiang, Jie Zhang, Wei Ren, Zuo-Guang Ye

**Affiliations:** 1 Electronic Materials Research Laboratory, Key Laboratory of the Ministry of Education & International Center for Dielectric Research, Xi’an Jiaotong University, Xi’an 710049, China; 2 Collaborative Innovation Center of High-End Manufacturing Equipment, Xi’an Jiaotong University, Xi’an,710049, China; 3 Energy Systems Division, Argonne National Laboratory, Lemont, IL 60439, USA; 4 State Key Laboratory for Manufacturing Systems Engineering, Xi’an Jiaotong University, Xi’an, 710049, China; 5 Department of Chemistry and 4D LABS, Simon Fraser University, Burnaby, British Columbia, V5A 1S6, Canada

## Abstract

Highly-ordered and conformal iron oxide nanotube arrays on an atomic scale are successfully prepared by atomic layer deposition (ALD) with controlled oxidization states and tunable magnetic properties between superparamagnetism and ferrimagnetism. Non-magnetic α-Fe_2_O_3_ and superparamagnetic Fe_3_O_4_ with a blocking temperature of 120 K are *in-situ* obtained by finely controlling the oxidation reaction. Both of them exhibit a very small grain size of only several nanometers due to the nature of atom-by-atom growth of the ALD technique. Post-annealing α-Fe_2_O_3_ in a reducing atmosphere leads to the formation of the spinel Fe_3_O_4_ phase which displays a distinct ferrimagnetic anisotropy and the Verwey metal-insulator transition that usually takes place only in single crystal magnetite or thick epitaxial films at low temperatures. The ALD deposition of iron oxide with well-controlled phase and tunable magnetism demonstrated in this work provides a promising opportunity for the fabrication of 3D nano-devices to be used in catalysis, spintronics, microelectronics, data storages and bio-applications.

The ever-increasing demand for highly ordered, conformal and magnetically-controlled nanostructures in spintronics^1^,^2^ and bio-applications^3^,^4^,^5^,^6^,^7^,^8^ has propelled the development of various methods for controlling the growth of such nanostructures within an atomic resolution. Atomic layer deposition (ALD) technique, featured with self-limiting surface reactions^9^, is ideal for the synthesis of these nanostructures due to its precise control of the thickness at the angstrom or monolayer level, excellent step coverage^10^ and conformal deposition on high aspect ratio structures^11^,^12^,^13^,^14^,^15^. As a matter of fact, ALD has already been accepted by the microelectronic industry as the key technique for preparing high-quality dielectrics for the trench capacitors in DRAMs^16^,^17^ and high dielectric constant gate oxides in CMOS transistors^18^,^19^. A typical ALD process consists of two or more different vapour-phase chemical reactions, in which several gaseous reactants are alternatively brought to the surface of the substrates and chemical reactions take place at the surface. Therefore, materials grown by ALD are, in principle, extremely smooth and conformal to the original substrates without the influence of the surface geometric complexity, which meets the essential requirements for the fabrication of magnetic nano-devices, such as magnetic tunneling junctions^20^ and memories^21^. However, the creation of magnetic nanostructures by ALD still remains challenging due to their complex binary or ternary chemical reactions as well as the difficulties in controlling the evolution of magnetic ordering. The monolayer atom-by-atom growth mechanism of ALD with a relatively low growth temperature often leads to a small grain size and poor polycrystallinity, which may restrict the spin interactions within a nano region, resulting in unusual magnetic orderings, such as ferrimagnetism and superparamagnetism.

Among various geometric types of objects^22^,^23^,^24^, tubular structure offers a high aspect ratio as compared to nanowires and nanoparticles, which introduces an additional degree of freedom in their designs, making it interesting for applications in catalysis^25^, spintronics, and biotechnologies. This high aspect ratio is expected to strongly influence the magnetic shape anisotropy and domain structure in individual tubes, which in turn leads to complex magnetic behaviors upon coupling with the static magnetic force in an ordered tube array. However, ultra-thin, homogeneous and smooth tubes with a wall thickness of several nanometers are difficult to be synthesized by conventional approaches. In bio-applications, such as cell separation and drug delivery, magnetic nanotubes exhibit a large drug loading efficiency, a strong magnetic field response and sharp penetration for the cell. Utilization of magnetic nanotubes in biomedical applications requires a superparamagnetic state with zero remnant magnetization in order to avoid the possible agglomeration^26^. Therefore, preparation of nanotubes with super-paramagnetism is essential for bio-applications, which still remains a central challenge. In this work, we have successfully synthesized highly-ordered iron oxide nanotube arrays with well-controlled phases, morphologies and magnetic properties through anodic aluminum oxide (AAO) template-assisted atomic layer deposition. Not only can the phase of the iron oxides be precisely controlled between Fe_3_O_4_ and α-Fe_2_O_3_ by finely adjusting the oxygen impulse period, but also the magnetic ordering can be tuned from the superparamagnetism to ferromagnetism through post-growth thermal treatment. The as-grown Fe_3_O_4_ nanotubes show well-defined superparamagnetic behavior with a blocking temperature of 120 K. This superparamagnetism is proposed to arise from the very small grain size achieved by the unique character of the atomic layer deposition, with atom-by-atom growth process at a relatively low temperature. The ferrimagnetic Fe_3_O_4_ nanotubes, exhibiting a distinct magnetic anisotropy and a Verwey metal-insulator transition, are obtained by annealing α-Fe_2_O_3_ in a mixture of 5% H_2_ and 95% Ar gases at 400 °C. It is worth mentioning that the Verwey metal-insulator transition, which usually only appears in single crystalline Fe_3_O_4_ or thick epitaxial Fe_3_O_4_ film, is found to occur in Fe_3_O_4_ nanotubes for the first time. The atomic layer deposition of Fe_3_O_4_ with well-controlled phases, morphologies and magnetic properties provides a viable platform for realizing conformal 3D magnetic nano-devices for potential applications in spintronics, electronics, data storage and bio-applications.

## Results and Discussions

**Figure 1:**
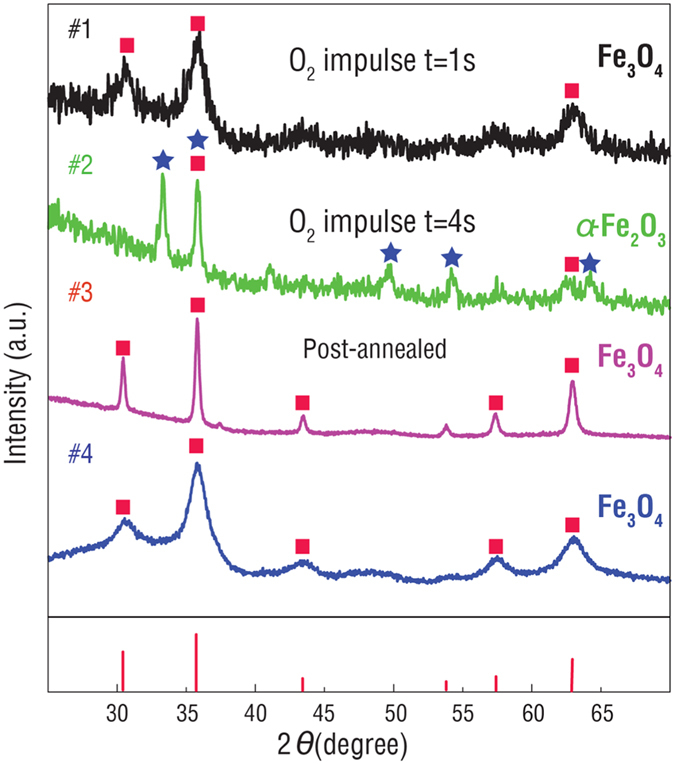
X-ray diffraction patterns of iron oxides prepared by various ALD processes, #1) As-grown Fe_3_O_4_ nanotubes with an O_2_ pulse duration of 1 second. #2) As-grown Fe_3_O_4_ nanotubes with an O_2_ pulse duration of 4 seconds. #3) Fe_3_O_4_ nanotubes obtained by post-annealing α-Fe_2_O_3_. #4) Fe_3_O_4_ nanotubes grown by the quasi-static mode.

Figure 1 shows the x-ray diffraction patterns of the iron oxide nanotube arrays prepared under various growth conditions. Pure Fe_3_O_4_ nanotubes are successfully obtained when the pulse time for oxygen is set for a short period of 1 second (#1). The peak positions and the intensity ratios suggest a polycrystalline nature of the Fe_3_O_4_ nanotubes without preferred orientation. However, the broadness of the diffraction peaks, as well as the low signal-to-noise ratio, indicates a poor crystallinity with a small grain size of 5 nm and possible presence of an amorphous phase. When the pulse duration of oxygen is set for a longer period of 4 seconds, the Fe_3_O_4_ phase is further oxidized and turns into a pure α-Fe_2_O_3_ phase, as shown in Fig. 1(#2). This demonstrates that the phase transition from the spinel Fe_3_O_4_ to α-Fe_2_O_3_ can be precisely controlled by adjusting the oxygen pulse duration. Upon further annealing α-Fe_2_O_3_ nanotube arrays in an atmosphere of 5% H_2_ and 95% Ar mixture for 3 hours at 400 °C, the α-Fe_2_O_3_ phase transforms back to Fe_3_O_4_ due to the reduction reaction. As a result, a typical XRD pattern of spinel Fe_3_O_4_ with a good (poly)crystallinity and high intensities is observed, as shown in Fig. 1(#3), and the narrow peaks indicate a large grain size (28.5 nm). In addition to varying the oxygen pulse, a quasi-static growth mode is also used to significantly enhance the diffusion of the Fe precursor into the AAO template. Quasi-static growth mode (enhanced growth model), which is used to significantly enhance the diffusion of the Fe precursor into the AAO template. In this mode, a high vacuum interrupt valve is installed between the reaction chamber and the vacuum pump, After the high vacuum interrupt valve switches off for 1s, the Fe precursor is pulsed into the reaction chamber and the high vacuum interrupt valve keeps close for 6s, so that the Fe precursor has sufficient time to diffuse into every corner of the AAO template for the thermal motion (The Fe precursor is pumped out through vacuum exhaust pipe once the source has been pulsed into the reaction chamber in the continued-flow mode.) This gives rise to a relatively long diffusion distance into the AAO template and more Fe atoms take part in the reaction. This growth mode allows the Fe to diffuse into the AAO template more homogenously, giving rise to more uniform and conformal nanotube arrays with a relatively good crystallinity, which is confirmed by the XRD pattern of Fe_3_O_4_ nanotube arrays grown by this mode (Fig. 1 #4). A better crystallinity is observed compared with the one grown by the continued-flow mode, and the broad diffraction peaks indicate a grain size of 6 nm, which is much smaller than that (28.5 nm) observed in the post-annealed Fe_3_O_4_ (The grain sizes of all the iron oxide nanotube arrays are calculated using the Scherrer equation based on the (311) peaks of the XRD patterns and given in Table S1 in Supporting Information). Therefore, by precisely controlling the growth conditions, iron oxides with desired phases of Fe_3_O_4_ and α-Fe_2_O_3_ and different grain sizes have been successfully synthesized by the ALD technique.

**Figure 2:**
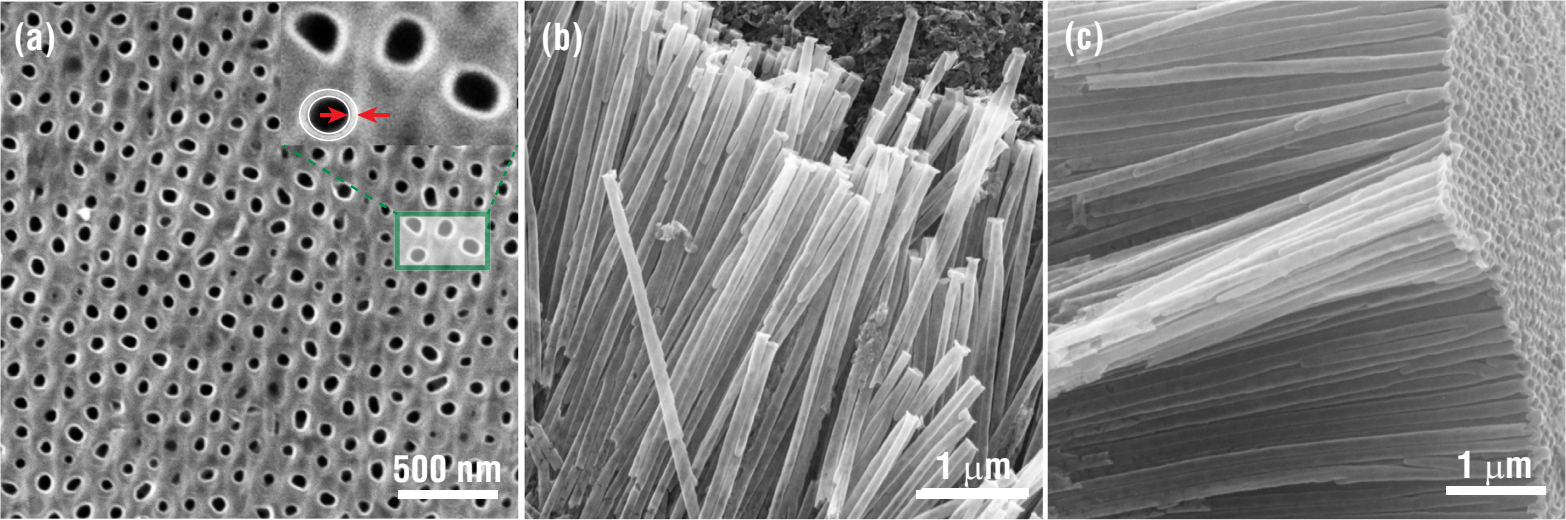
SEM images of the well-ordered Fe_3_O_4_ nanotubes obtained by ALD using the *in-situ* quasi-static mode. (**a**) Top view image of the Fe_3_O_4_ nanotubes deposited in the AAO template. (**b,c**) Free-standing and well-ordered Fe_3_O_4_ nanotubes arrays after removing the AAO template.

The morphology of the well-ordered Fe_3_O_4_ nanotube arrays prepared by the quasi-static growth mode is characterized using FESEM, as shown in Fig. 2. The cross-section features of the nanotubes grown in the AAO templates are presented in the top-view image (Fig. 2a). Prior to conducting SEM imaging, the sample is cleaned by ion milling to eliminate the metal oxide thin films on the surfaces. Uniform nanotube walls with a distinct contrast between Fe_3_O_4_ and Al_2_O_3_ are observed, indicating a smooth and conformal growth of Fe_3_O_4_ in the holes of the AAO templates. The individual tube thickness is determined to be 20 ± 2 nm (inset in Fig. 2a). Figures 2b,c show the well-ordered Fe_3_O_4_ nanotube arrays after the AAO template is completed removed. The free-standing nanotubes arrays possess a smooth and uniform surface without defects, such as cracks or pinholes. The tubes are open-end with a length larger than 30 μm, which is comparable to the physical dimensions of the AAO templates (More SEM images of the AAO template and open-end nanotube arrays are presented in Figs. S2 and S3 in Supporting Information)

**Figure 3:**
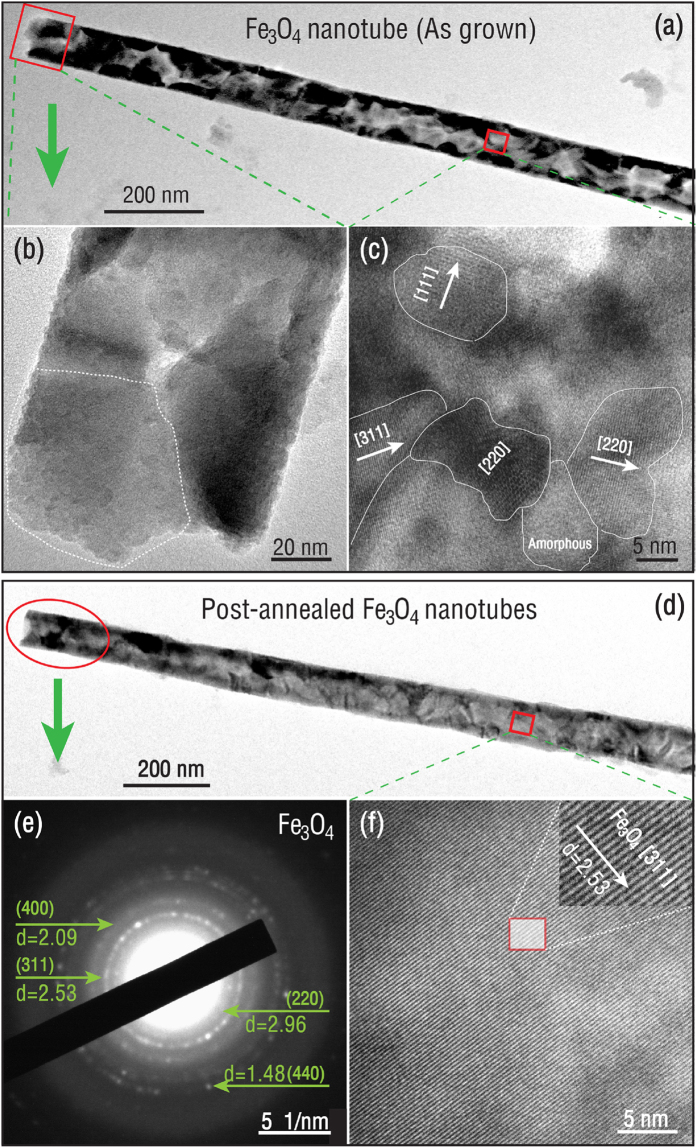
TEM and HRTEM images of an as-grown Fe_3_O_4_ nanotube (**a–c**) and a post-annealed Fe_3_O_4_ nanotube (**d–f**), and selected area electron diffraction pattern of the Fe_3_O_4_ nanotube (**e**).

Figure 3 shows the TEM and high resolution TEM (HRTEM) images of the as-grown and post-annealed Fe_3_O_4_ nanotubes on different scales. The as-grown Fe_3_O_4_ nanotube exhibits a large aspect ratio (Fig. 3a). Upon further zooming in selected areas, we find that the nanotube is made up of very small grains, indicating the polycrystalline nature of the as-grown Fe_3_O_4_ nanotubes (Fig. 3b). The average grain size is about 6 nm, which is consistent with the XRD result in Fig. 1(#1). Figure 3c shows the HRTEM image for a selected area in the Fe_3_O_4_ nanotube, which reveals the existence of small crystalline grains with different orientations. The indexes for these nano-grains correspond to a pure inverse spinel Fe_3_O_4_ phase. Some areas of an amorphous iron oxide phase are also observed surrounded by the nanocrystalline grains (More TEM images of the as-grown Fe_3_O_4_ nanotubes are presented in Figure S4 in Supporting Information). The formation of small grains is attributed to the atom-by-atom growth mechanism of ALD and the low deposition temperature. As shown in Fig. 3, the small grain size results in a superparamagnetic state. Figures 3d,e show the TEM image and the selected area electron diffraction (SAED) pattern of a post-annealed Fe_3_O_4_ nanotube, respectively. The SAED pattern confirms the spinel phase of the post-annealed Fe_3_O_4_ nanotubes (Fig. 3e), which is consistent with the XRD pattern in Fig. 1(#3). The HRTEM image in Fig. 3f reveals a large and homogenous area of the same orientation, indicating the grain size is dramatically enlarged and the crystallinity is significantly improved after post-annealing the nanotubes at a reducing atmosphere (More TEM images of the post-annealed Fe_3_O_4_ nanotubes are shown in Figure S5 in Supporting Information). It is expected that the large grain size and the polycrystallinity of the post-annealed Fe_3_O_4_ nanotubes may lead to an enhanced magnetism with a ferrimagnetic ordering and a strong magnetic anisotropy due to the large aspect ratio.

**Figure 4:**
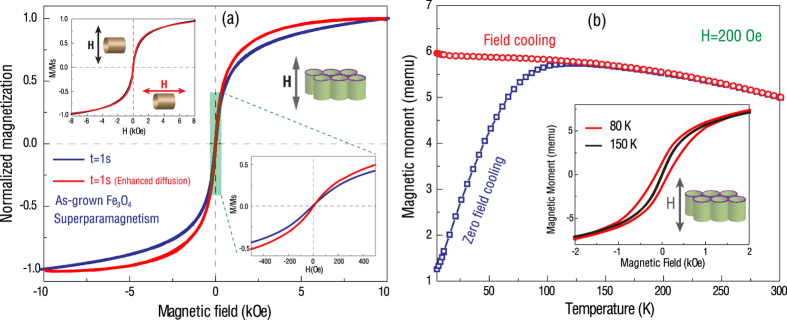
(**a**) Room temperature field-dependent magnetization curves of the as-grown Fe_3_O_4_ nanotube arrays prepared by the continued-flow mode and the quasi-static growth mode. The upper-left inset shows the non-linear *M*-*H* curves measured with the external fields parallel and perpendicular to the tube direction, respectively. (**b**) Zero-field-cooled (ZFC) and field-cooled (FC) magnetization curves as a function of temperature for the as-grown Fe_3_O_4_ nanotubes measured with an applied field of 200 Oe between 10 K and 300 K. The inset shows the hysteresis loop measured at 80 K and the non-hysteretic curve at 150 K.

In order to find out the influence of the growth conditions on the magnetic properties of the Fe_3_O_4_ nanotube arrays, we study the temperature dependence of magnetization as a function of applied magnetic field. Figure 4a shows the room temperature field-dependent magnetization (*M-H*) curves of the as-grown Fe_3_O_4_ nanotube arrays prepared by both the continued-flow mode and quasi-static growth mode. Both samples show a non-linear *M-H* curve with zero remnant magnetization, indicating a typical superparamagnetic behavior. It is believed that the small grain size (<6 nm) in the as-grown Fe_3_O_4_ inhibits the formation of long-range magnetic ordering, leading to the superparamagnetic ordering. This is confirmed by the fact that the *M-H* curves measured in the directions parallel and perpendicular to the tube length are identical, as shown in the upper-left inset of Fig. 4a. To investigate the competition between the thermal fluctuation and the magnetic interaction and to determine the transition temperature (or blocking temperature) from ferrimagnetism to superparamagnetism, the magnetization is measured as a function of temperature, as shown in Fig. 4b. When the as-grown Fe_3_O_4_ tube arrays are cooled down to 10 K at zero field, the electron spins are randomly distributed, showing a near-zero net moment. On the contrary, if the sample is cooled under a magnetic field of 1000 Oe, the electronic spins are well-aligned along the field direction, exhibiting a large net moment. Such zero-field-cooled (ZFC) and filed-cooled (FC) samples exhibit very different magnetic behaviors when measured upon heating under a bias field of 200 Oe. The ZFC sample shows a sharp increase in the magnetization from 10 K up to 120 K, indicating that the magnetic moments undergo a gradual rotation to align along the external magnetic field direction. In contrast, the FC sample exhibits an almost constant magnetization since most of the moments have been aligned along the external field direction. As the temperature reaches the blocking temperature *T*_B_ = 120 K, the two curves overlap each other, indicating a magnetic ordering transition from ferrimagnetism to superparamagnetism. Such magnetic transition is also confirmed by the change in the *M-H* behavior from an open loop measured at 80 K to a non-hysteretic curve at 150 K (inset of Fig. 4b).

**Figure 5:**
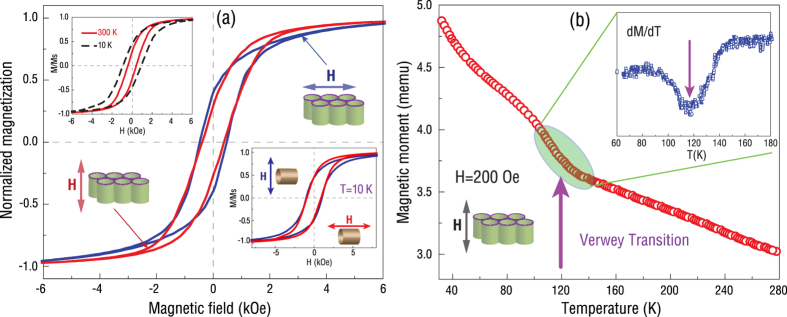
(**a**) Field-dependent magnetization curves of the post-annealed Fe_3_O_4_ nanotube arrays measured by applying external magnetic fields parallel and perpendicular to the tube direction, respectively, at room temperature. The upper inset is the hysteresis loops measured at 300 K and 10 K, respectively, with the magnetic field applied along the tube length direction. The lower-right inset shows the hysteresis loops measured parallel and perpendicular to the tube direction at 10 K. (**b**) Magnetic moment as a function of the temperature. The inset is the first-order derivative.

Figure 5 shows the magnetic properties of the post-annealed Fe_3_O_4_ nanotube arrays. The open hysteresis loops of magnetization as a function of magnetic field indicate a well-defined ferrimagnetic ordering at room temperature (Fig. 5a). Compared with the as-grown superparamagnetic Fe_3_O_4_ nanotubes, the annealing process not only turns α-Fe_2_O_3_ to Fe_3_O_4_, but also renders a larger grain size and good (poly)crystallinity, resulting in the ferrimagnetic ordering. The annealed Fe_3_O_4_ nanotube arrays exhibit a distinct magnetic anisotropy with the magnetic easy axis parallel to the tube length direction and the hard axis perpendicular to the tube direction at both room and low temperatures (lower-right inset of Fig. 5). As the temperature decreases, the coercive field increases, which is consistent with what was observed in the thin film or bulk Fe_3_O_4_ (upper-left inset of Fig. 5a). Figure 5b shows the magnetization as a function of the temperature, measured with the external magnetic field applied parallel to the tube direction. It is known that magnetite possesses a metal-insulator transition (Verwey transition) upon cooling at T_V_ = 125 K, which is known to be extremely sensitive to stoichiometry deviations as well as impurity content^27^. Upon cooling to the magnetic phase transition temperature T_V_, the crystal structure transforms from the cubic inverse spinel (T > T_V_) to a monoclinic structure (T < T_V_), which is accompanied by an abrupt increase in resistivity, coupled to a slight change in magnetic moment^28^. The mechanism behind the Verwey transition is the subject of a long-standing debate between the charge ordering model (‘Wigner crystal’) of the Fe^2+^/Fe^3+^ cations on the octahedral sites and the structural/orbital ordering model, and remains an active topic of research. The Verwey transition is usually observed in single crystal bulks or thick films of Fe_3_O_4_. In annealed Fe_3_O_4_ nanotubes, we observe a slight change in magnetic moment in the temperature range of 110 ~130 K, which seems to indicate the Verwey metal-insulate transition. The temperature dependence of the relative magnetization change (dM/dT) shows a clear anomaly close to 117 K (inset of Fig. 5b), confirming the phase transition. To the best of our knowledge, this is the first observation of the Verwey transition in Fe_3_O_4_ nanotube arrays. The appearance of the Verwey transition indicates a high-quality of the magnetite nanotubes.

**Figure 6:**
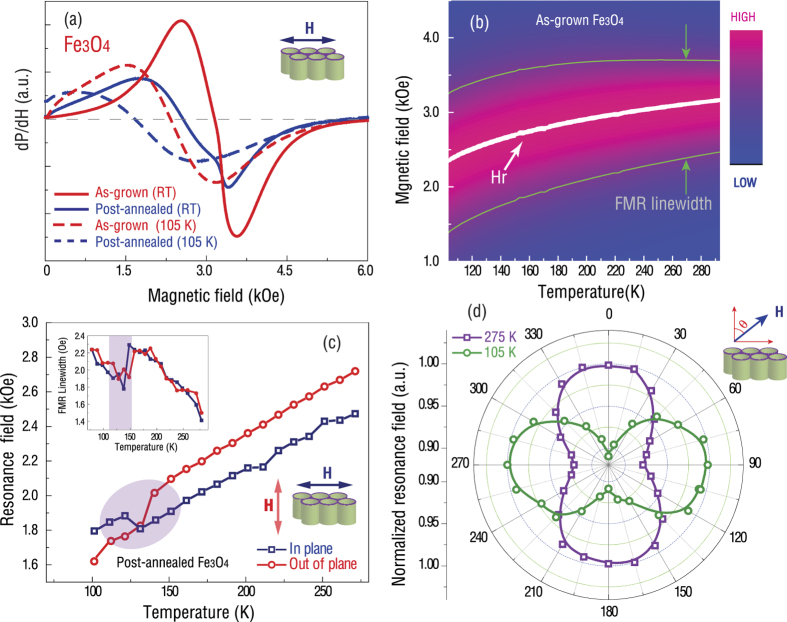
(**a**) EPR spectra of the as-grown and post-annealed Fe_3_O_4_ nanotubes measured at 275 K and 105 K (at 9.3 GHz). The external magnetic field is perpendicular to the tube length direction. (**b**) The variations of the resonance filed *H*_*r*_ and the corresponding FMR linewidth deduced from the EPR spectra as a function of temperature for the as-grown Fe_3_O_4_ nanotubes. (**c**) The resonance field as a function of temperature for the post-annealed Fe_3_O_4_ nanotubes, with the external magnetic fields parallel and perpendicular to the tube length direction. The upper inset is the FMR linewidth as a function of temperature. (**d**) Angular dependences of the normalized resonance fields at 275 K and 105 K.

In order to investigate the spin dynamics and to quantitatively determine the magnetization variation near the Verwey transition, the electron paramagnetic resonance (EPR) spectra are measured for both the as-grown and post-annealed Fe_3_O_4_ nanotube arrays. Figure 6a presents the EPR spectra of as-grown and post-annealed Fe_3_O_4_ nanotubes measured at 105 K and 275 K. A typical superparamagnetic EPR behavior is found in the as-grown sample with a resonance field of 3360 Oe (measured at 9.3 GHz) at room temperature. For the post-annealed ferrimagnetic Fe_3_O_4_ nanotubes, the ferromagnetic resonance (FMR) absorption appears at a lower magnetic field of 2200 Oe, which is close to the resonance field of the ferromagnetic Fe_3_O_4_ thin film^17^. Figure 6b shows the variations of the resonance filed *H*_*r*_ and the corresponding FMR line width deduced from the EPR spectra as a function of temperature for the as-grown Fe_3_O_4_ nanotubes. Upon cooling the sample, the resonance field drops gradually without displaying any distinct transition from superparamagnetic to ferrimagnetic ordering, while the FMR linewidth becomes broader. Figure 6c shows the variation of the FMR field as a function of temperature for the post-annealed ferrimagnetic Fe_3_O_4_ nanotubes measured with the external magnetic field applied parallel and perpendicular to the tube directions, respectively. Though an overall linear correlation is observed, sudden changes in the resonance field take place near 120 K for both curves, which is consistent with the observed phase transition shown in Fig. 5b. Meanwhile, the FMR linewidth as a function of temperature also displays an abrupt change near the Verwey transition temperature, as shown in the inset of Fig. 6c. These results further confirm that the Verwey transition indeed takes place in the post-annealed Fe_3_O_4_ nanotubes at 120 K, which alters the magnetic anisotropy and magnetization, resulting in a distinct change in the resonance field. Figure 6d shows the angular dependence of the resonance field for the post-annealed sample field at 300 K and 100 K. A strong magnetic shape anisotropy is found in ferrimagnetic Fe_3_O_4_ nanotube arrays, where the magnetic easy axis is along the tube length direction at the temperature above 120 K. However, when the temperature drops below the transition temperature, the magnetic anisotropy is switched by 90°, with the easy axis becoming perpendicular to the tube length direction. We relate this phenomenon to the Verwey transition-induced structural change which leads to the variation of magnetic anisotropy and the switching of the magnetic easy and hard axes.

## Conclusions

Well-ordered iron oxide nanotube arrays with controlled phase and tunable magnetic properties are fabricated by atomic layer deposition. With fine control of the growth conditions and the oxidization level, as-grown α-Fe_2_O_3_ and Fe_3_O_4_ nanotube arrays are obtained with very small grains of 6 nm size, showing non-magnetic and superparamagnetic properties, respectively, with a blocking temperature of 120 K for the latter. When annealed in a mixture of H_2_/Ar gases at 400 ^o^C, the α-Fe_2_O_3_ turns to the ferrimagnetic Fe_3_O_4_ phase by reduction reactions. It is interesting to note that the Verwey transition, which is usually observed only in a single crystal Fe_3_O_4_, is found to take place for the first time in Fe_3_O_4_ nanotubes formed by ALD. The successful deposition of iron oxides by ALD with controlled oxidation states and different types of magnetic ordering provides a viable platform for realizing conformal 3D magnetic nano-devices with potential applications in spintronics, microelectronics, data storage and bio-sensors.

## Methods

Iron oxide nanotubes are prepared by ALD with the assistance of anodic aluminum oxide (AAO) templates. Ferrocene (Fe(Cp)_2_, 99.9%) and oxygen gas (O_2_, 99.999%) are used as the precursors for iron and oxygen, respectively. Alternating pulses of these precursors are introduced into the ALD reactor chamber sequentially by purging with carrier gas of N_2_. The deposition temperature is 400 °C and the flow of carrier gases is set at 100 sccm and 200 sccm for ferrocene and oxygen, respectively. During the growth, the pulse duration of injecting ferrocene precursor remains constant (0.4 second). However, the pulse duration of oxygen is varied to control the oxidization level (The schematics of deposition of iron oxide nanotube arrays are shown in Figure S1 in Supporting Information). Individual nanotubes are obtained by removing the AAO template in a NaOH solution of 1 mol/L. The morphology and microstructure of the nanotubes are characterized by field emission scanning electron microscope (FESEM), transmission electron microscope (TEM) and x-ray diffraction (XRD). The magnetic properties of the nanotube arrays are investigated by a superconducting quantum interference device (SQUID) and an electron paramagnetic resonance (EPR) spectrometer.

## Additional Information

**How to cite this article**: Zhang, Y. *et al.* Controlled Phase and Tunable Magnetism in Ordered Iron Oxide Nanotube Arrays Prepared by Atomic Layer Deposition. *Sci. Rep.* **6**, 18401; doi: 10.1038/srep18401 (2016).

## Supplementary Material

Supplementary Information
