# Supplementary material for: Controlled Phase and Tunable Magnetism in Ordered Iron Oxide Nanotube Arrays Prepared by Atomic Layer Deposition
Source: Sci Rep. 2016 Jan 27;6:18401. doi: 10.1038/srep18401 (PMC4728408; doi:10.1038/srep18401)
Supplement: Supplementary Information [file srep18401-s1.pdf]

**[Supplementary Information]**

# Controlled phase and tunable magnetism in ordered iron oxide nanotube arrays prepared by atomic layer deposition

Yijun Zhang[1], Ming Liu[1,2*], Bin Peng[1], Ziyao Zhou[3], Xing Chen[3], Shu-Ming Yang[2,4], Zhuang-De Jiang[2,4], Jie Zhang[1], Wei Ren[1,2*], Zuo-Guang Ye[1,2,5*]

[1] *Electronic Materials Research Laboratory, Key Laboratory of the Ministry of Education & International Center for Dielectric Research, Xi'an Jiaotong University, Xi'an 710049, China*

[2] Collaborative Innovation Center of High-End Manufacturing Equipment, Xi'an Jiaotong University, Xi'an, 710049, China

[3] *Energy Systems Division, Argonne National Laboratory, Lemont, IL 60439, USA*

[4.] *State Key Laboratory for Manufacturing Systems Engineering, Xi'an Jiaotong University, Xi'an, 710049, China*

[5] *Department of Chemistry and 4D LABS, Simon Fraser University, Burnaby, British Columbia, V5A 1S6, Canada*

**Table S1** The calculated grain size of the iron oxide nanotube arrays using the Scherrer formula based on the (311) peaks of the XRD spectra in Figure 1.

| Sample No | #1 | #2 | #3 | #4 |
|---|---|---|---|---|
| Grain Size | 5 nm | 29 nm | 28.5 nm | 6 nm |

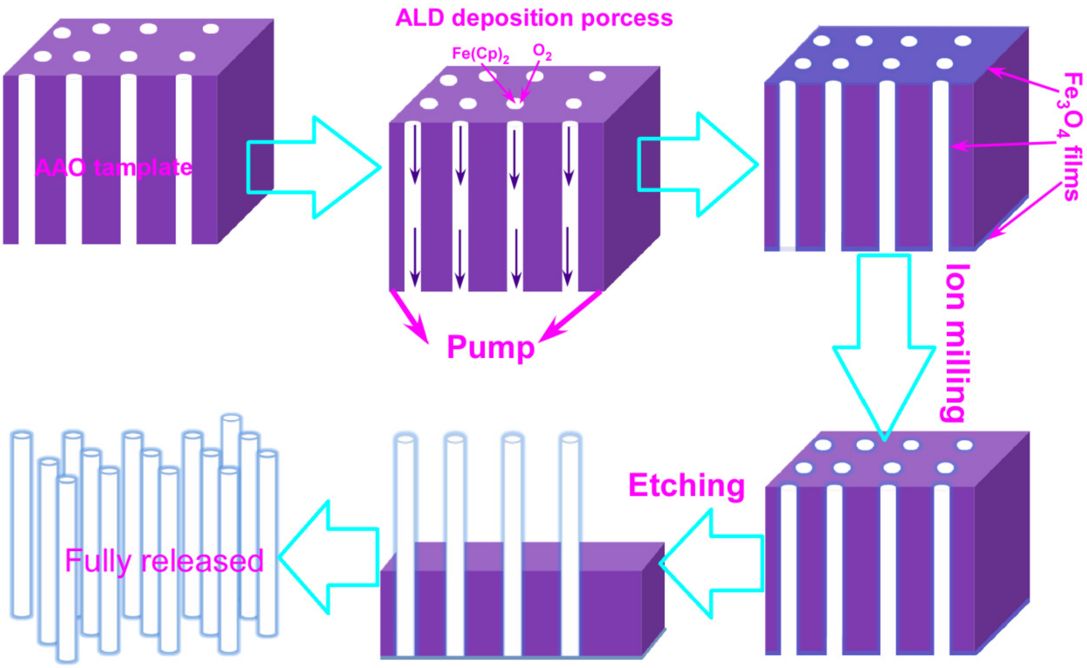


Figure S1. The schematic of $Fe_3O_4$ nanotube arrays fabrication process by ALD.

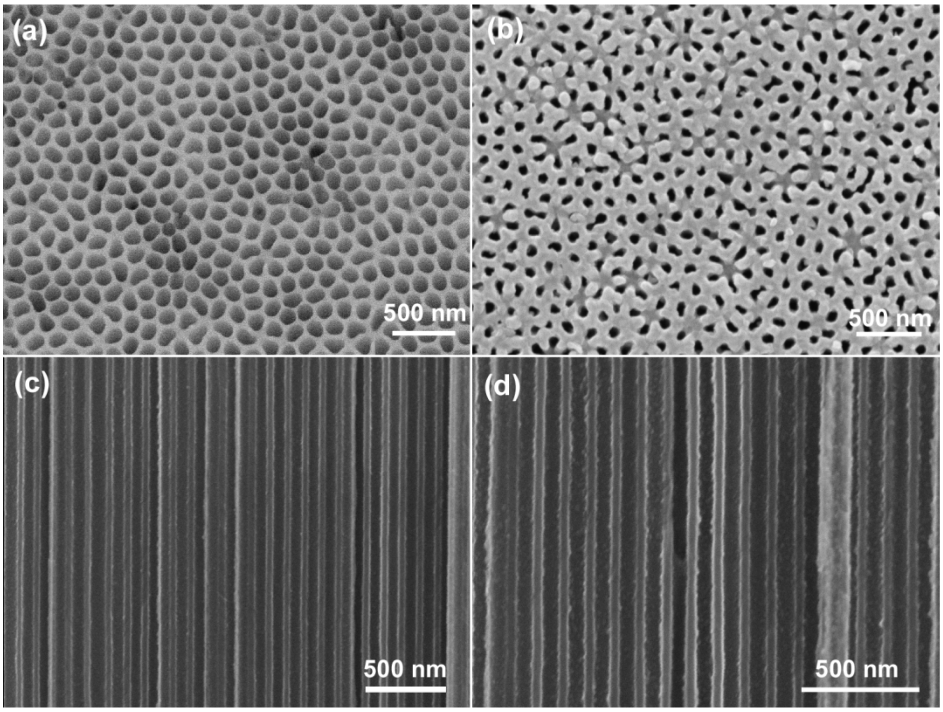


Figure S2. The SEM images of the top view (a) and cross section(c) of the blank AAOtemplate; the SEM images of the top view (a) and cross section(c) after the $Fe_3O_4$ thin films have been grown on the template by the in situ quasi-static mode.

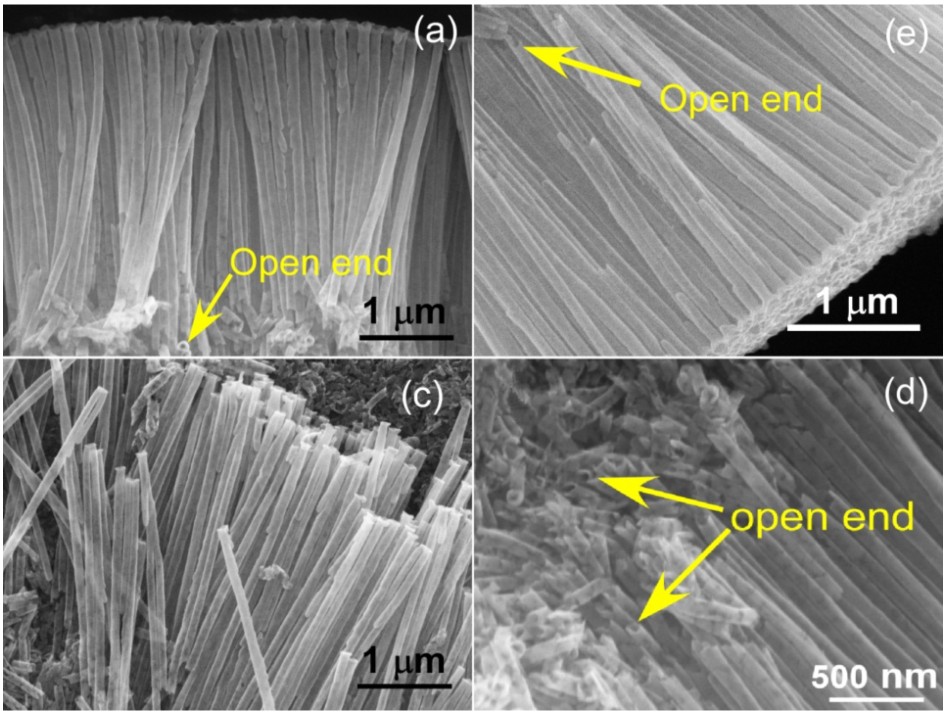


Figure S3. SEM image of the well-ordered $Fe_3O_4$ nanotube obtained by the in situ quasi-static mode, side view images of the well-ordered $Fe_3O_4$ nanotube arrays (a) and (b). To clearly show the hollow nature of the $Fe_3O_4$ nanotube arrays, the man-made broken end of the $Fe_3O_4$ nanotubes are shown in (c) and (d).

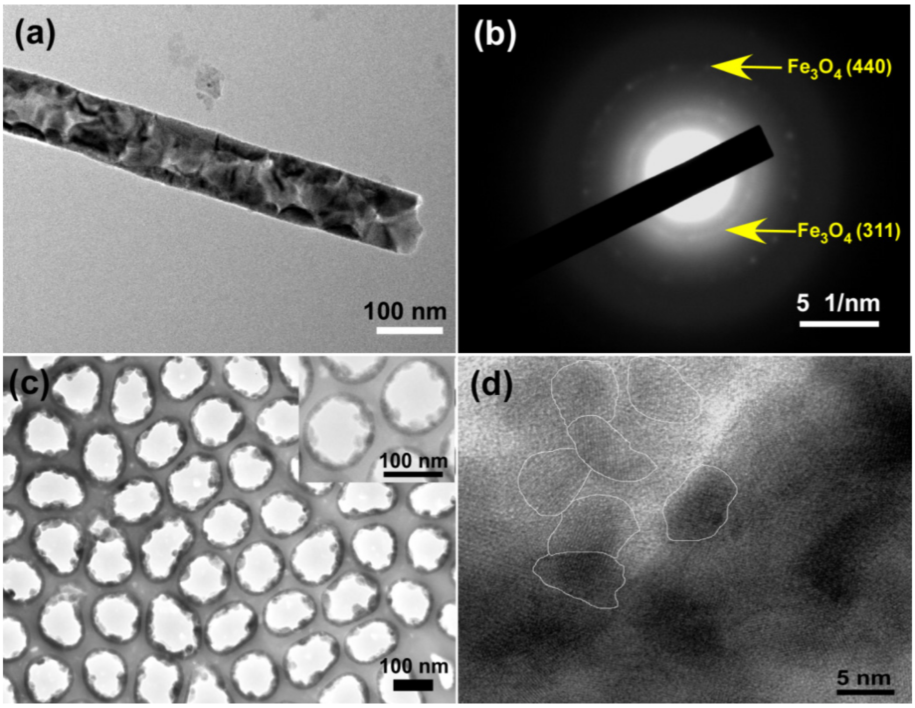


Figure S4. TEM images of an as-grown $Fe_3O_4$ nanotube by enhanced mode with the low magnification morphology image (a), the selected area electron diffraction pattern (b), the crossection image of the $Fe_3O_4$ nanotube in the AAO template (c) and the high resolution TEM image of $Fe_3O_4$ nanotube (d).

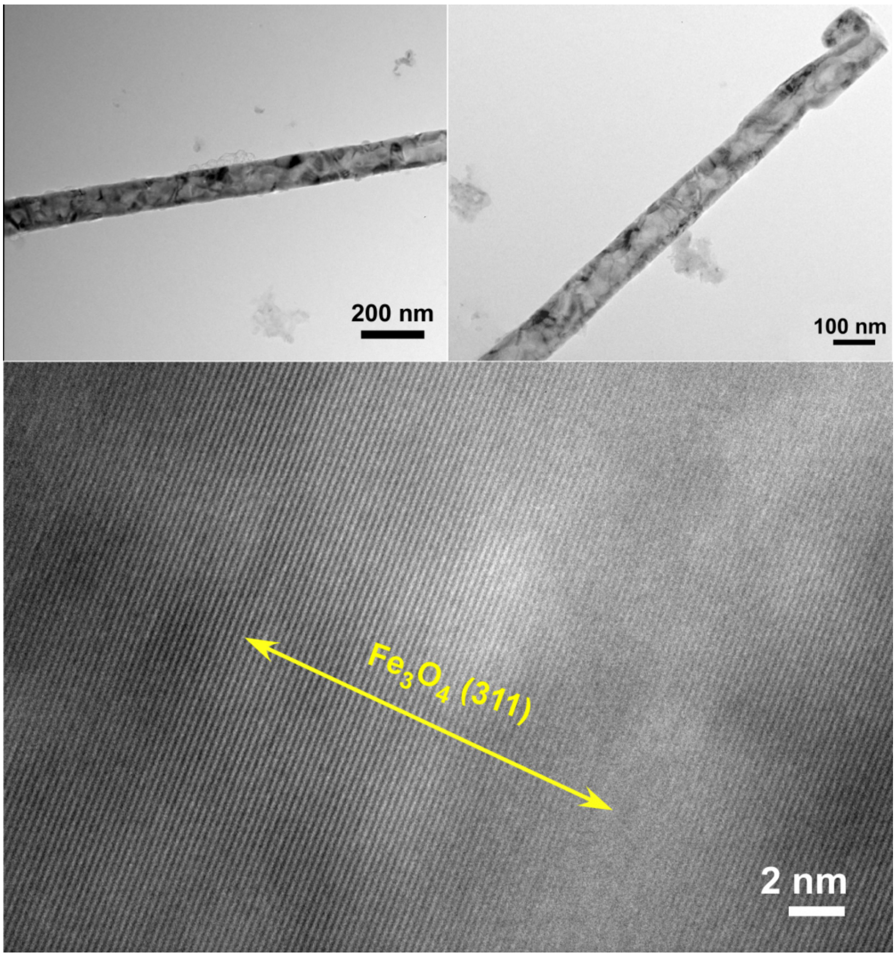


Figure S5. TEM images of the $Fe_3O_4$ nanotube obtained by post-annealing an α-$Fe_2O_3$ nanotube in reductive atmosphere.
